# Micropropagation and Subsequent Enrichment of Carotenoids, Fatty Acids, and Tocopherol Contents in *Sedum dasyphyllum* L

**DOI:** 10.3389/fchem.2017.00077

**Published:** 2017-10-09

**Authors:** Han Yong Park, Ramesh Kumar Saini, Judy Gopal, Young-Soo Keum, Doo Hwan Kim, Onew Lee, Iyyakkannu Sivanesan

**Affiliations:** ^1^Department of Bioresource Engineering, Sejong University, Seoul, South Korea; ^2^Department of Bioresources and Food Science, Konkuk University, Seoul, South Korea

**Keywords:** carotenoids, cytokinin, gas chromatography, fatty acids, mass spectrophotometry, α-tocopherol, shoot multiplication

## Abstract

A promising micropropagation protocol has been systematically established and demonstrated for the enhanced production of carotenoids, tocopherol and fatty acids in shoot tissues of *Sedum dasyphyllum*. Shoot tip explants were grown on Murashige and Skoog (MS) medium. Different concentrations of N^6^-benzyladenine (BA) or thidiazuron (TDZ) alone or in combination with α-naphthaleneacetic acid (NAA) were tested in order to stimulate multiple shoot production. Ideal shoot induction (100%) and maximized shoot numbers (36.4) were obtained on explants cultured on media incorporated with 2 μM BA and 1 μM NAA combinations. The *in vitro*-developed shoots rooted best on half-strength MS media incorporated with 2 μM indole 3-butyric acid. Plantlets were effectively acclimatized in the greenhouse with 100% survival rate. The composition and contents of bioactive compounds such as carotenoids, tocopherol and fatty acids in shoot tissues of *S. dasyphyllum* were investigated using HPLC and GC-MS. The most abundant carotenoid in the shoot tissue was all-*E*-lutein (40.3–70.5 μg g^−1^ FW) followed by 9′-*Z*-neoxanthin (5.3–9.9 μg g^−1^ FW), all-*E*-violaxanthin (4.4–8.2 μg g^−1^ FW), and all-*E*-β-carotene (1.6–3.6 μg g^−1^ FW). The α-tocopherol contents of *in vitro*-raised shoots was 6.5-fold higher than shoots of greenhouse-grown plants. The primary fatty acids found in shoot tissues were α-linolenic acid (32.0–39.3%), linoleic acid (27.4–38.2%), palmitic acid (13.3–15.5%), and stearic acid (5.2–12.2%). In all, summarizing the findings, the micropropagated *S. dasyphyllum* showed significant enrichment of valuable bioactive carotenoids (92.3 μg g^−1^ FW), tocopherols (14.6 μg g^−1^ FW), and α-linolenic acid (39.3%) compared to their greenhouse counterparts. The protocol demonstrated here could be applied for the mass propagation and production of enhanced bioactive compounds from *S. dasyphyllum* with credibility.

## Introduction

The genus *Sedum* L., from the family Crassulaceae, commonly known as “stonecrops” comprises of about 600 species most of which are distributed in the Northern Hemisphere. Most of the *Sedum* plants are edible and have been used in traditional medicine against various diseases (Xu et al., 2015). Several *Sedum* species are cultivated as ornamental plants because of their attractive look and hardiness. Pharmacological investigations have revealed that *Sedum* species possess antiangiogenic, anticholinesterase, anti-inflammatory, antimicrobial, antinociceptive, antioxidant, antitumor, and hepatoprotective properties (He et al., 1998; Bonina et al., 2000; Kim et al., 2004; De Melo et al., 2005; Jung et al., 2008; Van Diermen et al., 2010; Dahpour et al., 2012; Ertas et al., 2014; Bensouici et al., 2016; Chen et al., 2016). Phytochemical investigations reveal the presence of several medical compounds in *Sedum* species like alkaloids, chlorogenic acid, chrysin, coumarins, cyanogenic glycoside, cyclotrisiloxane, eicosane, flavonoids (hesperetin, kaempferol, naringenin, and quercetin), hexadecanoic acid, neophytadiene, phenolic acids (caffeic and ferulic), and terpenes (Bonina et al., 2000; De Melo et al., 2005; Yoshikawa et al., 2007; Van Diermen et al., 2009; Dahpour et al., 2012; Morikawa et al., 2012; Beltrán-Orozco et al., 2013).

*Sedum dasyphyllum* L., commonly known as Corsican stonecrop, is designated as a rare medicinal plant of the Mediterranean region (Agelet and Vallès, 2001). In folk medicine, the aerial parts of *S. dasyphyllum* have been used for treating wounds in the Iberian Peninsula (Rigat et al., 2011) and possess anti-algic, anti-inflammatory and antiseptic properties (Agelet et al., 2000; Agelet and Vallès, 2001; Rigat et al., 2011). It is reported to contain caffeic acid, cyanogenic glycoside, ferulic acid, flavonols, flavonoid glycosides, and isoflavones (Yoshikawa et al., 2007). Antioxidant and cancer chemopreventive activities were reported previously (Van Diermen et al., 2009, 2010). Asexual propagation is a useful method for multiplication of *Sedum* species. However, low proliferation rate and the shortage of plant materials often affected their mass cultivation. Thus, *in vitro* propagation methods have been developed in several *Sedum* species for mass multiplication and plant improvement (Brandao and Salema, 1977; Kitamura et al., 2002; Yoon et al., 2002; Wojciechowicz, 2007, 2009; Zhao et al., 2009; Yang et al., 2012; Kim and Sivanesan, 2016; Liu et al., 2017). In these studies, leaves, nodes, petals, shoot tips and stem explants obtained from greenhouse- or field- grown plants of *Sedum acre, S. aizoon, S. gracile, S. floriferum, S. alfredii, S. drymarioides, S. erythrostichum, S. plumbizincicola, S. sarmentosum, S. spectabile*, and *S. telephium* were used for shoot or plant regeneration. The authors have studied the impact of 2,4-dichlorophenoxyacetic acid (2,4-D), N^6^-benzyladenine (BA), gibberellic acid (GA_3_), indole 3-acetic acid (IAA), indole 3-butyric acid (IBA), α-naphthaleneacetic acid (NAA), and thidiazuron (TDZ) on callus induction, organogenesis, shoot proliferation, shoot elongation, somatic embryogenesis and root induction.

The establishment of *in vitro* cell and organ cultures of *S. dasyphyllum* will be useful for secondary metabolite production. Several bioactive compounds like alkaloids, carotenoids, fatty acids, flavonoids, phenolic acids and tocopherols were obtained from *in vitro* shoot cultures of important plants (Jeong and Sivanesan, 2015, 2016; Sivanesan et al., 2016a,b; Dowom et al., 2017; Grzegorczyk-Karolak et al., 2017). Till date, no published reports are available on the micropropagation of *S. dasyphyllum*. The objectives of the present work were (1) to investigate the impact of BA or TDZ alone or in synergy with NAA on multiple shoot production from shoot tip explants of *S. dasyphyllum*, (2) to evaluate the effects of IAA or IBA on *in vitro* rooting of shoots, and (3) to compare and contrast the enhancement in bioactive compounds in shoots of *in vitro*-raised against greenhouse-grown plants.

## Materials and methods

### Plant material, reagents, and standards

Shoot tips of *S. dasyphyllum* collected from 2-year-old greenhouse-grown plants were used for the micropropagation study. For bioactive compound analysis, 4-month-old shoots of *S. dasyphyllum* were obtained from *in vitro*-raised shoot cultures and greenhouse-grown plants. Murashige and Skoog (MS) medium, BA, IAA, IBA, NAA, TDZ, sucrose and plant agar were purchased from Duchefa Biochemie, Haarlem, The Netherlands. Standard of all-*E*-lutein was procured from Cayman Chemical Company, Michigan, USA. 9′-*Z*-neoxanthin and all-*E*-violaxanthin were bought from DHI LAB products Hoersholm, Denmark. All-*E*-β-carotene, fatty acid standard mix (CRM47885- Supelco 37 Component FAMES Mix), certified reference material (BCR-485) and α-tocopherol, were purchased from Sigma-Aldrich, St. Louis, MO, USA. All organic solvents used for extraction of bioactive compounds were of HPLC grade (Daejung, Korea).

### The micropropagation protocol

Actively growing shoots of *S. dasyphyllum* collected from 2-year-old greenhouse-grown plants were thoroughly washed under running water and rinsed with sterile distilled water. The shoots were surface sterilized with 70% (v/v) ethanol for 60 s and 2.5% (v/v) sodium hypochlorite containing few drops of Tween 20 for 10 min. This was followed by five washes with sterile distilled water, then blot dried using sterile filter paper to remove traces of water. Shoot tips (about 1.0 cm long) isolated from the sterilized shoots were cultured on MS medium (Murashige and Skoog, 1962) amended with different concentrations and combinations of BA, TDZ and NAA for shoot multiplication (Tables 1, 2). The experiment was conducted in triplicates with 25 explants for each treatment. The frequency of shoot induction and the number of shoots were recorded after a culture period of 8 weeks. The shoot induction percentage was calculated as the number of explants developing shoots divided by a total number of explants cultured × 100. The regenerated shoots were maintained on MS liquid or semisolid medium containing 2 μM BA and 1 μM NAA and subcultured at 8 week intervals. For root induction, *in vitro*-raised shoots (1.0–1.5 cm) were cultured on half-strength MS medium augmented with 0, 1, 2, or 4 μM IAA or IBA (Table 3). The experiment was conducted in triplicates with 50 shoots for each treatment. The frequency of root induction, root numbers and length was recorded after a culture period of 5 weeks. The root induction percentage was calculated as the number of shoots rooted divided by a total number of inoculated shoots × 100. The culture medium consisted of MS nutrients and vitamins amended with 3% (w/v) sucrose and 0.8 % (w/v) plant agar. The pH of the medium was adjusted to 5.6, 100 ml of the medium was dispensed in plant culturing container (103 × 78.6 mm), and each container was closed with a lid (101 × 102 mm, Phytohealth, SPL Life Sciences, Korea), and autoclaved at 121°C for 20 min. The culture containers were maintained at 25 ± 2°C under a 16-h photoperiod with a photosynthetic photon flux density of 45 μmol m^−2^ s^−1^. Rooted shoots were transplanted into pots containing peat moss, perlite and vermiculite (1:1:1, v/v/v), and maintained in a culture room. Plantlets were fertigated with quarter-strength MS salts solutions every 4 days, and the survival was recorded after a period of 5 weeks.

**Table 1:** Effects of BA and TDZ on shoot multiplication from shoot tip explants of *S. dasyphyllum*.

**BA (μM)**	**TDZ (μM)**	**Shoot induction (%)**	**Number of shoots**
0	0	45.2 ± 3.1f	3.8 ± 0.7e
1	0	71.6 ± 3.8e	13.6 ± 1.7b
2	0	93.4 ± 3.6a	18.4 ± 1.0a
4	0	84.4 ± 3.1c	10.6 ± 1.9c
8	0	81.2 ± 3.3d	8.8 ± 1.2cd
0	1	89.2 ± 2.3b	12.0 ± 2.1bc
0	2	85.0 ± 2.0c	10.2 ± 1.2c
0	4	82.6 ± 3.3cd	7.6 ± 1.4d
0	8	70.8 ± 2.8e	6.2 ± 1.5d

*Means ± SD within a column followed by different letters (a–f) are significantly different using DMRT based on p < 0.05*.

**Table 2:** Effects of combination of BA and NAA on shoot multiplication from shoot tip explants of *S. dasyphyllum*.

**BA (μM)**	**NAA (μM)**	**Shoot induction (%)**	**Number of shoots**
1	1	92.0 ± 2.8b	27.2 ± 1.7b
2	1	100 ± 0.0a	36.4 ± 2.7a
1	2	74.2 ± 2.4d	13.2 ± 1.7cd
2	2	89.8 ± 3.7c	16.0 ± 1.7c
1	4	63.2 ± 2.6e	11.4 ± 1.5d
2	4	50.8 ± 2.7f	7.2 ± 1.2e

*Means ± SD within a column followed by different letters (a-f) are significantly different using DMRT based on p < 0.05*.

**Table 3:** Rooting response of *in vitro* produced shoots of *S. dasyphyllum* cultured on half-strength MS medium containing 3% (w/v) sucrose with different concentrations of IAA and IBA.

**IAA (μM)**	**IBA (μM)**	**Root induction (%)**	**Number of roots**	**Root length (cm)**
0	0	95.6 ± 1.9b	6.4 ± 1.0e	2.4 ± 0.2c
1	0	100 ± 0.0a	6.8 ± 1.2e	3.6 ± 0.5b
2	0	100 ± 0.0a	10.2 ± 2.0c	4.1 ± 0.2b
4	0	100 ± 0.0a	8.0 ± 0.9d	4.4 ± 0.4b
0	1	100 ± 0.0a	11.2 ± 2.1bc	5.2 ± 0.4ab
0	2	100 ± 0.0a	19.8 ± 2.8a	6.6 ± 0.4a
0	4	100 ± 0.0a	13.4 ± 2.1b	6.1 ± 0.5a

*Means ± SD within a column followed by different letters (a–e) are significantly different using DMRT based on p < 0.05*.

### Bioactive compounds analysis

The extraction and quantification of carotenoids and tocopherols was done following Rodriguez-Amaya (2001) and Saini and Keum (2017) protocols with slight modifications. All the preparations were performed in low light conditions to avoid the light-mediated degradation. Briefly, 2.0 g finely chopped fresh shoots were transferred into an amber glass vial, homogenized with 10 ml of cold acetone having 0.1% (w/v) butylated hydroxytoluene (BHT). The supernatant was collected subsequent to centrifugation at 5,000 rpm for 5 min. Pelleted samples were continually extracted until total decoloration was evident. The supernatant of each extraction was combined, vacuum-dried on a rotary evaporator at 35°C, dissolved in 5 ml of cold acetone containing 0.1% (w/v) BHT, filtered through a syringe filter (0.45 μm, Whatman), and finally transferred to an amber colored vial for HPLC analysis. Carotenoid and tocopherol analysis was carried out using an HPLC instrument (Agilent Model 1100, Agilent Technologies Canada Inc., Mississauga, ON, Canada) furnished with an autosampler, degasser, diode array detector (200–800 nm) and binary pump, and separated using C30 column (YMC), 250 × 4.6 mm, 5 μm (YMC, Wilmington, NC). The column thermostat was maintained at 20°C temperature. The solvent system consisted of methanol: methyl tertiary butyl ether: water (81:15:4) (eluent A) and methyl tertiary butyl ether: methanol (91:9) (eluent B). The gradient elution consisted of 0–50% B for 45 min, followed by 0% B and 5 min post run at a flow rate of 1 ml/min. The detection wavelengths were 295 and 450 nm for tocopherol and carotenoids, respectively. The injection volume was 20 μl. Quantitative determinations of carotenoids and tocopherols was done by comparing the dose-response curves created from authentic standards. The Purity of the purified fractions was determined by HPLC (i.e., a chromatogram showing a single peak). The percentage of purity was calculated as the percentage of the carotenoid and tocopherol peak area relative to total area (Kimura and Rodriguez-Amaya, 2002). The HPLC analytical method used for quantification of carotenoids and tocopherols was validated in terms of linearity. For each compound, calibration (standard) curves were constructed by plotting the peak area against the six-different standard concentration within the working range, and the correlation coefficient was determined (US Food Drug Administration, 2001). The purity, working range and correlation coefficient of standards are represented in Table 4.

**Table 4:** The purity, working range and correlation coefficient of authentic standards of carotenoids and tocopherol.

**Authentic standards**	**% Purity of standard**	**Working range (μg/ml)**	**Correlation coefficient (*R*^2^)**
All-*E*-violaxanthin	96.5	0.15–10	0.998
9′-*Z*-Neoxanthin	97.3	0.15–10	0.997
All-*E*-Lutein	95.0	0.15–10	0.999
All-*E*-Zeaxanthin	98.0	0.15–10	0.999
All-*E*-β-Carotene	97.0	0.15–10	0.996
α-tocopherol	95.2	3.0–100	1.000

The lipids in shoot tissues of *S. dasyphyllum* were extracted as described by Bligh and Dyer (1959) and Saini and Keum (2017) with slight modifications. Briefly, 2.0 g finely chopped fresh shoots were transferred into an amber glass vial, homogenized with 20 ml of chloroform: methanol (2:1 v/v), and then the supernatant was collected after centrifugation (5,000 rpm) for 5 min at 4°C. Pelleted samples were continually extracted until decoloration. The supernatant from each extraction was combined in a separating funnel (250 ml) and partitioned with 30 ml of 0.85% sodium chloride. The chloroform phase (lower) was collected into a pre-weighted glass tube, dried on a rotary evaporator under reduced pressure, and the total lipid content was determined gravimetrically. FAMEs were prepared and analyzed by GC-MS (GC-2010 Plus Gas Chromatograph (Shimadzu, Japan) equipped with AOC-20 i Autoinjector and GCMS-QP2010 SE Gas Chromatograph-mass spectrophotometer using a slightly polar RXi-5Sil column (Restek; 30 m × 250 μm id × 0.25 lm film). Injector port and the detector temperatures were set up at 250° and 230°C, respectively. Helium was used as the carrier gas. Firstly, column temperature was maintained at 120°C for 5 min, followed by increasing the temperature to 240°C in 30 min using a linear temperature program of 4°C/min and held at 240°C for 25 min (Sivanesan et al., 2016a). The FAMEs were identified by comparing their fragmentation pattern and retention time with authentic standards and also with the database from the National Institute Standard and Technique (NIST)/Environmental Protection Agency (EPA)/National Institutes of Health (NIH) Mass Spectral Database (NIST08 and NIST08S) Library (Saini et al., 2014).

### Data analysis

For GC and HPLC analysis, all the samples were extracted in triplicates and analyzed separately in duplicates. Values from all six determinations of each sample were averaged and represented as means with standard deviation (SD). The experimental results were subjected to analysis of variance using an SAS program and expressed as the mean ± SD. The differences between the average values were assessed by Duncan's multiple range test (DMRT) based on *p* < 0.05.

## Results

### Accreditation of the micropropagation methodology of *S. dasyphyllum*

Multiple shoots were obtained from shoot tip explants of *S. dasyphyllum* grown on Murashige and Skoog (MS) medium in the presence and absence of phytohormones. However, shoot induction frequency and the average number of shoots significantly varied among different treatments (Table 1). On cytokinin-free medium, 45.2% explants responded and produced a mean of 3.8 shoots. The supplementation of BA or TDZ significantly enhanced the shoot induction percentage and shoot numbers. The frequency of shoot induction and shoot number improved as the concentration of BA in the culture medium increased from 0 to 2 μM and then declined with further increase in BA levels. The maximum frequency of shoot induction (93.4%) with a mean of 18.4 shoots was obtained on MS medium incorporated with 2 μM BA (Table 1). Of the different levels of TDZ tested, 1 μM TDZ induced maximum frequency of shoot induction (89.2%) with an average of 12 shoots per explant. However, an increasing level of TDZ above 1 μM decreased both the percentage of shoot formation and shoot numbers. Among the two cytokinins applied in this study, BA was found to be ideal for multiple shoot production than TDZ. Thus, BA at 1 and 2 μM was selected for further experiments.

**Figure 1:**
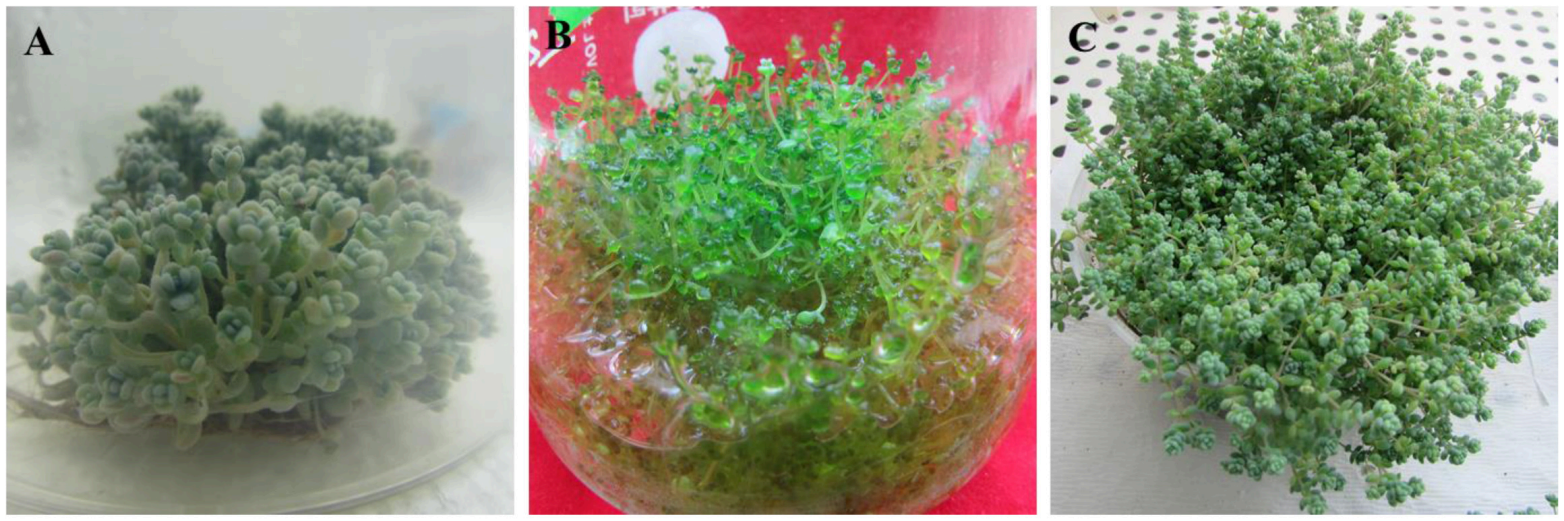
Micropropagation of *S. dasyphyllum*. **(A)** Shoot tip explants cultured on MS semisolid medium incorporated with 2 μM BA and 1 μM NAA; **(B)** Shoot tip explants grown on MS liquid medium fortified with 2 μM BA and 1 μM NAA; **(C)** Acclimatized plants.

The BA and NAA combo significantly improved both the frequency of shoot formation and shoot numbers (Table 2). The highest shoot induction percentage (100%) and maximum shoots (36.4) were obtained when the shoot tips were grown on MS medium incorporated with 2 μM BA and 1 μM NAA (Figure 1A). However, optimal levels of BA (2 μM) combined with higher concentrations of NAA (2 and 4 μM) significantly decreased the number of shoots produced per explant. On these media, the explants developed more callus than shoots. The *in vitro*-raised shoots were separated from the cluster and cultured on MS liquid or semisolid medium incorporated with 2 μM BA and 1 μM NAA for 8 weeks. The number of shoots and shoot lengths were significantly higher in liquid medium than the semisolid medium (Figure 1B, data not shown). However, the shoots were maintained well (6-month) on semisolid medium than in liquid cultures (2-month).

In this study, shoots acquired from MS medium containing 2 μM BA and 1 μM NAA developed roots in half-strength MS medium augmented with 0–4 μM IAA or IBA within 14 days of culture. Moreover, significant differences were observed in an average number of roots amidst the treatments (Table 3). The addition of IAA and IBA to the half-strength MS medium significantly increased the mean number of root and root length compared to the control. Optimal root induction response was observed on half-strength MS medium incorporated with 2 μM of IAA and IBA. However, increasing concentration of IAA or IBA above 2 μM decreased the number of roots developed per shoot. Of the two auxins, IBA was found to be ideal for *in vitro* rooting of *S. dasyphyllum* than IAA. The highest number of roots (19.8) and root lengths (6.6 cm) were obtained on media incorporated with 2 μM IBA. Interestingly the survival rate of the rooted plantlets (100%) was unaffected by the rooting media (Figure 1C).

### Characterizing the bioactive compound in shoots

**Figure 2:**
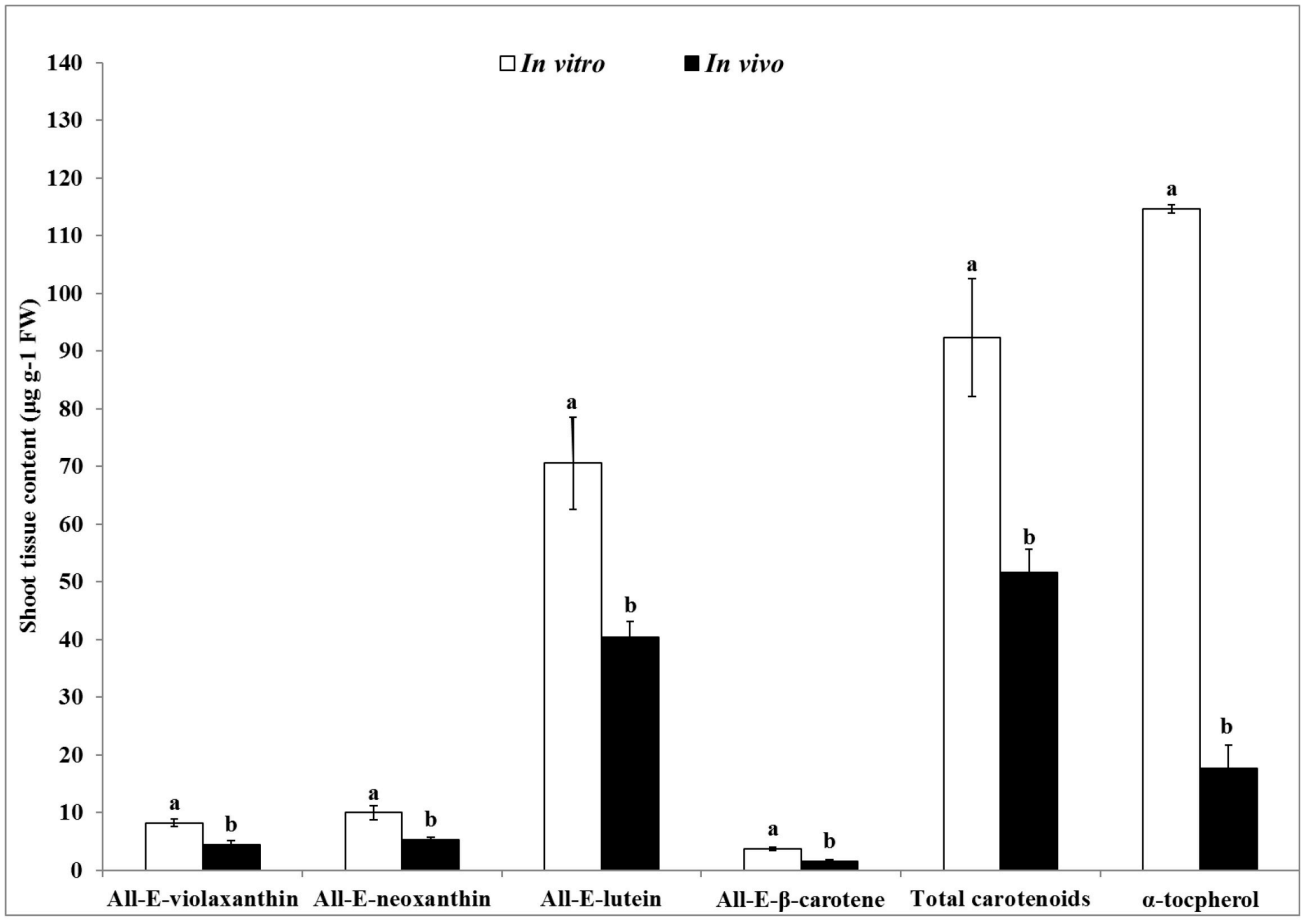
Carotenoids and α- tocopherol content in shoot tissues of *S. dasyphyllum*. Means ± SD followed by different letters (a,b) are significantly different using DMRT based on *p* < 0.05. Carotenoids and α-tocopherol were detected at 450 and 295 nm, respectively.

**Figure 3:**
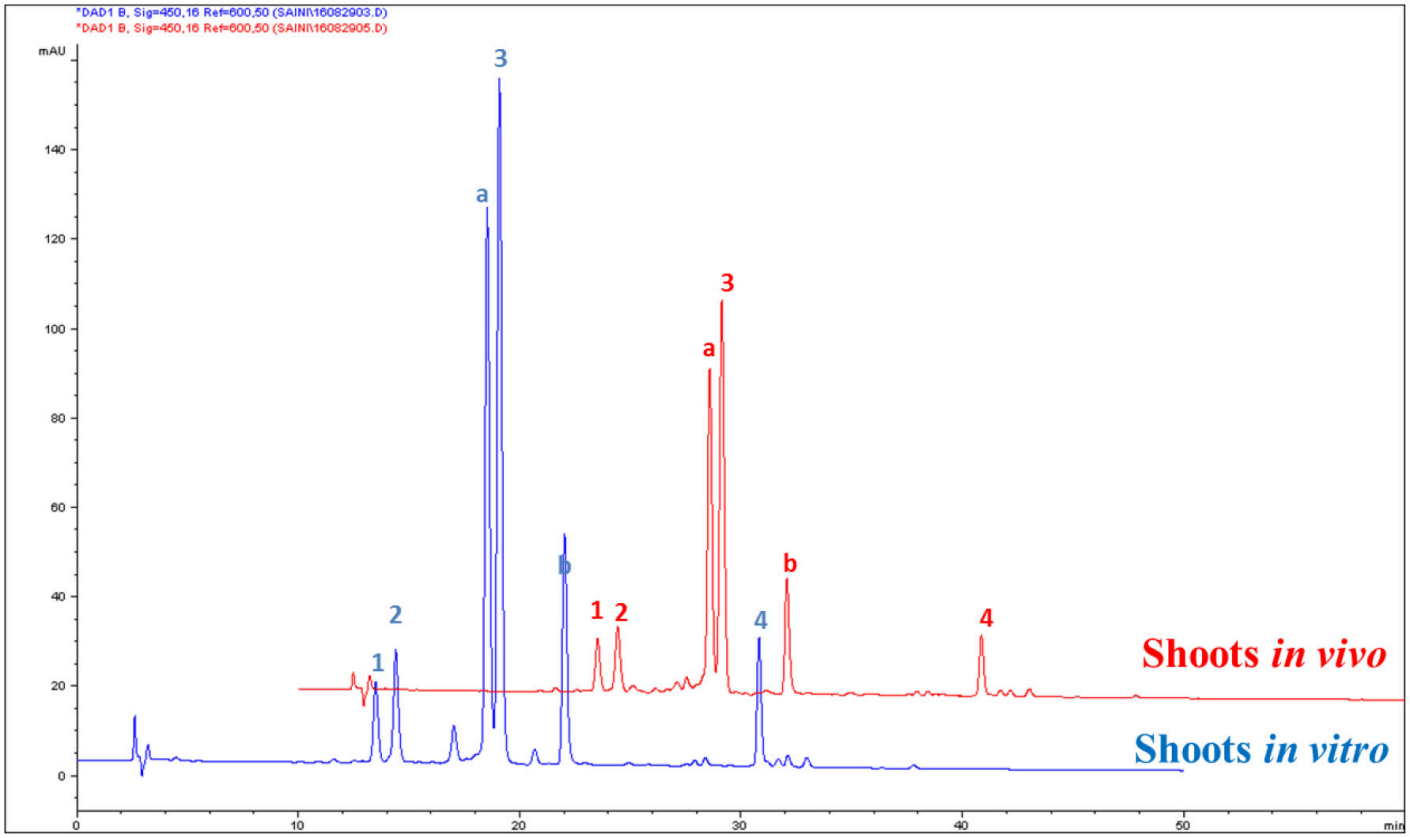
HPLC chromatograms (UV, 450 nm) of carotenoids in shoot tissues of *S. dasyphyllum*. 1. All-*E*-violaxanthin; 2. 9′-*Z*-neoxanthin; 3. All-*E*-lutein; 4. All-*E*-β-carotene; Peak a and b are chlorophylls (not quantified).

**Figure 4:**
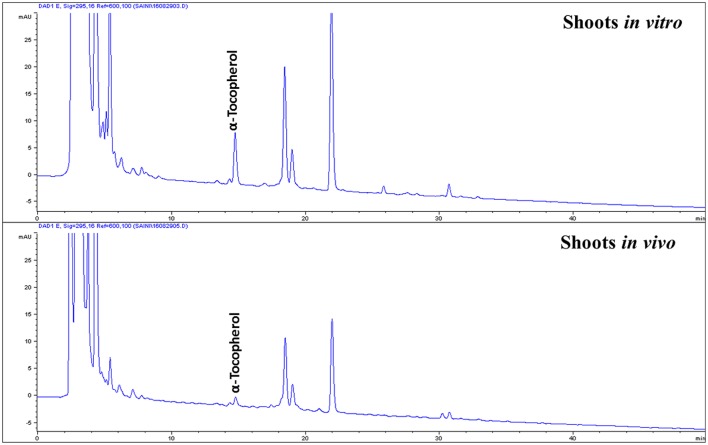
HPLC chromatograms (UV, 295 nm) of tocopherols in shoot tissues of *S. dasyphyllum*.

The contents of carotenoids and α-tocopherol in shoot tissues of *S. dasyphyllum* are represented in Figure 2. High-performance liquid chromatography (HPLC) analysis confirmed the presence of four major carotenoids such as all-*E*-violaxanthin, 9'-*Z*-neoxanthin, all-*E*-lutein and all-*E*-β-carotene in shoot tissues obtained from *in vitro*- and *in vivo*-grown plants (Figure 3). The most abundant carotenoid in shoot tissues of *S. dasyphyllum* was all-*E*-lutein (40.3–70.5 μg g^−1^ fresh weight (FW) followed by 9′-*Z*-neoxanthin (5.3–9.9 μg g^−1^ FW), all-*E*-violaxanthin (4.4–8.2 μg g^−1^ FW), and all-*E*-β-carotene (1.6–3.6 μg g^−1^ FW). The content of all-*E*-lutein in shoots (*in vitro*) and shoots (*in vivo*) were 70.5 and 40.3 μg g^−1^ FW, respectively (Figure 2). HPLC analysis further confirmed the presence of α-tocopherol in shoot tissues of *S. dasyphyllum* (Figure 4), while the other form of tocopherols were not detected in both shoots. The α-tocopherol content in the the *in vitro*-raised shoots were 6.5-fold higher than shoots of greenhouse-grown plants (Figure 2).

**Figure 5:**
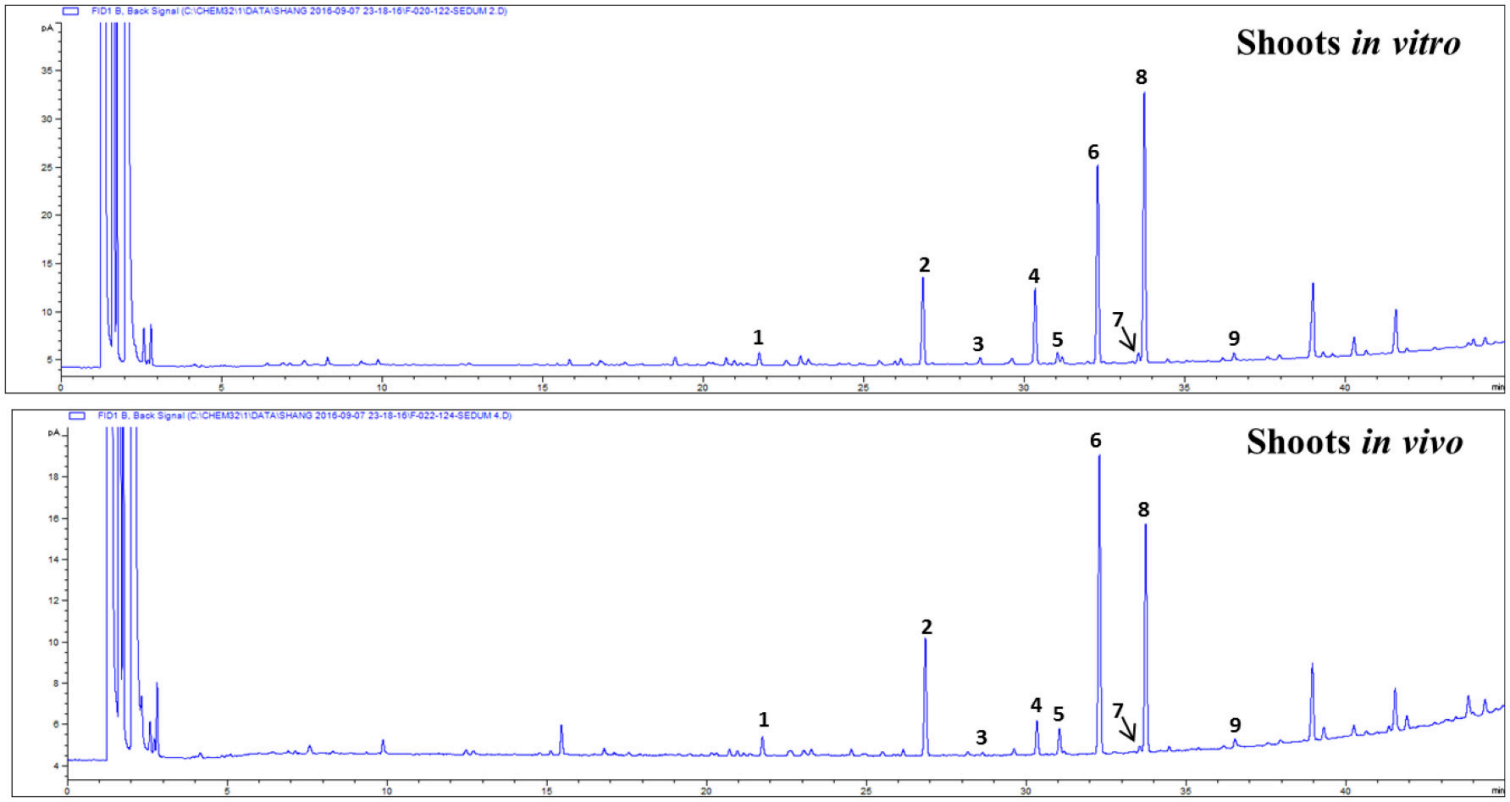
GC chromatogram of FAMEs in shoot tissues of *S. dasyphyllum*. 1. RT 21.793 myristic acid (C14:0), 2. RT 26.865 palmitic acid (C16:0), 3. RT 28.632 heptadecanoic acid (C17:0), 4. RT 30.335 stearic acid (C18:0), 5. RT 31.053 oleic acid (C18:1n9c), 6. RT 32.305 linoleic acid (C18:2n6c), 7. RT 33.536 arachidic acid (C20:0), 8. RT 33.773 α-linolenic acid (C18:3n3), 9. RT 36.489 behenic acid (C22:0).

Gas chromatography (GC)-mass spectrophotometry (MS) analysis revealed the presence of nine fatty acids in shoots of *S. dasyphyllum* (Figure 5, Table 5). There was no difference in the constituents of fatty acid methyl esters (FAMEs) in the shoot samples; however, the composition percentage of individual fatty acids varied between the samples. Alpha-linolenic acid (39.3%) was the most abundant fatty acid in regenerated shoots (*in vitro*) followed by linoleic acid (27.4%), palmitic acid (13.3%), stearic acid (12.2%), myristic acid (2.2%), oleic acid (1.6%), arachidic acid (1.5%), behenic acid (1.2%), and heptadecanoic acid (1.2%). In shoots obtained from the greenhouse-grown plants, the most rich fatty acid was linoleic acid (38.2%) followed by α-linolenic acid (32%), palmitic acid (15.5%), stearic acid (5.2%), oleic acid (3.4%), myristic acid (2.5%), behenic acid (1.7%), arachidic acid (0.9%), and heptadecanoic acid (0.8%). It was additionally observed that the level of polyunsaturated fatty acids (PUFA) was higher than the quantity of monounsaturated fatty acids (MUFA) in shoot tissues of *S. dasyphyllum* (Table 5). Further, maximum total lipid contents were also obtained from *in vitro* shoot cultures (3.5%) rather than the greenhouse specimens (1.9%).

**Table 5:** Composition of fatty acids in shoot tissues of *S. dasyphyllum*.

**Fatty acids**	**Retention time**	**Shoots (*in vitro*)**	**Shoots (*in vivo*)**
C14:0 Myristic acid (SFA)	21.793	2.2 ± 0.16b	2.5 ± 0.09a
C16:0 Palmitic acid (SFA)	26.865	13.3 ± 0.68b	15.5 ± 0.11a
C17:0 Heptadecenoic acid (SFA)	28.632	1.2 ± 0.18a	0.8 ± 0.04b
C18:0 Stearic acid (SFA)	30.335	12.2 ± 0.59a	5.2 ± 0.49b
C18:1n9c Oleic acid (MUFA)	31.053	1.6 ± 0.02b	3.4 ± 0.04a
C18:2n6c Linoleic acid (PUFA)	32.305	27.4 ± 0.74b	38.2 ± 1.23a
C20:0 Arachidic acid (SFA)	33.536	1.5 ± 0.11a	0.9 ± 0.05b
C18:3n3 α- Linolenic acid (PUFA)	33.773	39.3 ± 0.20b	32.0 ± 0.94a
C22:0 Behenic acid (SFA)	36.489	1.3 ± 0.19b	1.7 ± 0.05a
Σ SFA		31.6 ± 0.97a	26.5 ± 0.25b
Σ MUFA		1.6 ± 0.02b	3.4 ± 0.04a
Σ PUFA		66.8 ± 0.95b	70.2 ± 0.29a
PUFA: SFA		2.1 ± 0.09b	2.7 ± 0.04a
PUFA: MUFA		41.2 ± 0.05a	20.9 ± 0.32b
Total lipids		3.5 ± 0.11a	1.9 ± 0.17b

*Values are percentages of the total fatty acids, from the mean of triplicate extractions and analyses. Means ± SD followed by different letters (a,b) are significantly different using DMRT based on p < 0.05. SFA, saturated fatty acids; MUFA, monounsaturated fatty acids; and PUFA polyunsaturated fatty acids*.

## Discussion

### Micropropagation of *S. dasyphyllum*

Micropropagation is an efficient technique for mass production of reputed ornamental and medicinal plants (Jeong and Sivanesan, 2016; Kim and Sivanesan, 2016; Kwaśniewska and Pawłowska, 2017). Shoot tips and stem nodes are frequently used as explants for the multiplication of true-to-type plants. Cytokinins (BA or TDZ) have significant effects on multiple shoot production from shoot tips of *S. dasyphyllum*. Similarly, the addition of phytohormones to MS medium is reported to increase shoot production in *S. alfredii* (Liu et al., 2017). On the contrary, our previous reports have shown that the inclusion of BA or TDZ to MS medium did not influence shoot numbers in *S. sarmentosum* (Kim and Sivanesan, 2016). Thus, it appears that phytohormone requirements for ideal axillary shoot multiplication is species dependent and varies between different *Sedum* species. In this study, multiple shoots were best obtained in the presence of BA than TDZ (Table 1). Our results well correlate to the existing knowledge, that BA has been observed to be the most frequently used cytokinin for shoot induction in *Sedum acre, S. aizoon, S. gracile, S. floriferum* and *S. spectabile* (Wojciechowicz, 2007, 2009), *S. alfredii* and *S. plumbizincicola* (Zhao et al., 2009; Liu et al., 2017), *S. drymarioides* (Kitamura et al., 2002), *S. erythrostichum* (Yoon et al., 2002), and *S. telephium* (Brandao and Salema, 1977). Inhibitory effects at higher concentrations of TDZ on shoot production have also been reported earlier in *S. sarmentosum* (Kim and Sivanesan, 2016) and *S. spectabile* (Yang et al., 2012).

Shoot multiplication is controlled by the ratio of cytokinin and auxin. It has also been reported that the presence of higher concentrations of cytokinin and low concentration of auxins were effective in enhancing shoot multiplication, while the reverse, reduced shoot number and induced callus formation or root induction (Brandao and Salema, 1977; Kitamura et al., 2002; Wojciechowicz, 2007, 2009; Zhao et al., 2009; Yang et al., 2012). In this study, the greatest number of shoots produced on media amended with higher levels of BA and lower levels of NAA has been clearly demonstrated (Table 2). A combination of BA and IBA or NAA has often been used to maximize shoot formation in *Sedum* species (Brandao and Salema, 1977; Kitamura et al., 2002; Yoon et al., 2002; Wojciechowicz, 2007, 2009; Zhao et al., 2009; Liu et al., 2017). Successful *in vitro* rooting of shoots frequently depends on plant species, culture medium strength, and auxin treatment. Auxin is usually added to the culture medium to promote root induction in regenerated shoots of *S. alfredii* (Zhao et al., 2009) and *S. sarmentosum* (Kim and Sivanesan, 2016). However, *in vitro*-raised shoots of *Sedum acre, S. aizoon, S. gracile, S. floriferum*, and *S. spectabile* were rooted on auxin-free medium (Wojciechowicz, 2007). In this study, rooting of shoots was best achieved on media incorporated with 2 μM IBA (Table 3). Plantlets were effectively acclimatized in the greenhouse with 100% survival rate.

### Characterizing the bioactive compound in shoots

Carotenoids and tocopherols are important lipophilic antioxidants that are essential for animals, as well as man and plants (Rizvi et al., 2014; Esteban et al., 2015). In literature, the available data on the composition and contents of carotenoids and tocopherol in organs of *Sedum* is highly limited. For the first time, we have systematically delved into the carotenoids and tocopherol contents of *S. dasyphyllum*. The total carotenoid contents in shoots raised *in vitro* was more than double that of the greenhouse grown ones. A similar result has been previously reported in case of *Ajuga multiflora* also (Sivanesan et al., 2016a). However, yet another report showed that the content of total carotenoids was significantly greater in leaves obtained from greenhouse-grown plants than leaves collected from *in vitro*-raised shoots of *Aronia melanocarpa* (Sivanesan et al., 2016b). The content of α-tocopherol in the regenerated shoots (114.6 μg g^−1^ FW) of *S. dasyphyllum* was significantly higher (17.6 μg g^−1^ FW) compared to that obtained from greenhouse-grown plants. *In vitro* explant cultures are known to produce and accumulate several valuable bioactive compounds in enhanced quantities. The accumulation of bioactive compounds in plant cultures *in vitro* mostly depends on culture media composition and culture environments (Jeong and Sivanesan, 2015, 2016). It has been reported that carotenoids and tocopherols play a significant role in the defense mechanisms (Esteban et al., 2015). The addition of phytohormones like auxin and cytokinins to the growth medium resulted in stressful conditions and thereby probably increased shoot production. Such shoots can accumulate more amounts of bioactive compounds than the field- or greenhouse-grown plants. Moreover, nutrients required for the synthesis of metabolites are copiously enriched in the culture medium. In addition to this, several studies have already proved that the contents of the bioactive compounds in shoot cultures of *Bacopa monnieri* (Praveen et al., 2009), *Canscora decussate* (Kousalya and Bai, 2016), *Jeffersonia dubia* (Jeong and Sivanesan, 2016), *Nothapodytes nimmoniana* (Dandin and Murthy, 2012), *Rosa rugosa* (Jang et al., 2016), *Scrophularia takesimensis* (Jeong and Sivanesan, 2015), and *Silybum marianum* (Khan et al., 2014) were significantly much higher than field-grown plants. It is also reported that the auxins added to the adventitious root culture media, at lower or higher concentrations, not only regulate *in vitro* morphogenesis processes but also increase phenolic acid and triterpenoid saponin accumulation (Kikowska et al., 2014). It is probably due to the modification of the secondary metabolite biosynthesis pathway by those plant hormones as predicted by earlier workers in this area; that we see this enhancement of bioactive compounds in the micropropagated *Sedum* plants (Baque et al., 2010; Amoo et al., 2012; Baskaran et al., 2014; Moyo et al., 2014). Thus, a corroboration of (i) the effect of plant growth hormones, affecting the biosynthetic pathways and also resulting in stress induction; (ii) availability of adequate nutrients and (iii) presence of other physical, chemical and biological elicitors appears to have led to the manifold enhancement of the bioactive compounds in the micropropagated *Sedum* plants compared to their greenhouse-grown counterparts. More studies in this direction will help arrive at conclusive evidences.

The level of α-tocopherol in the *in vitro*-grown shoots of *S. dasyphyllum* was also higher compared to the hypocotyl (11.4 μg g^−1^ FW), stem (7.3 μg g^−1^ FW), leaf (18.1 μg g^−1^ FW), calli (19.8 μg g^−1^ FW), and cell suspension cultures (24.0 μg g^−1^ FW) of sunflower, and also greater than total tocopherols in cereals (17–60 μg g^−1^ FW), fruits (1.1–84 μg g^−1^ FW), legumes (4.8–16.7 μg g^−1^ FW) and vegetables (1.0–30 μg g^−1^ FW) as reported earlier (Caretto et al., 2010). Carotenoids and tocopherols are widely used in food and pharmaceutical industries due to their antioxidant and inflammatory roles. In this study, lutein and α-tocopherol were higher in shoot tissues of *S. dasyphyllum* than other bioactive compounds analyzed. Lutein is a desirable ingredient for several food products and also included in animal and fish feed. Alpha-tocopherol is one of the active ingredients in many food products. Several studies have shown that α-tocopherol may play an important role in the prevention and treatment of Alzheimer's disease, arthritis, atherosclerosis, cancer, cataracts, heart disease, and enhances humoral and cell immune responses (Rizvi et al., 2014). Fatty acids are used in food and pharmaceutical industries. In this study, the micropropagated shoots showed enhanced (39.3%) alpha-linolenic acid, while the greenhouse-grown plants possessed merely 32%. Alpha-linolenic acid (omega-3 fatty acid) holds an edge over linoleic acid (omega-6 fatty acid), in that it is recommended that one focuses more on increasing the omega-3 fat intake than your omega-6 intake (Green and Hilditch, 1935; McCutcheon, 1942). Omega-3 fatty acid has been reported to be beneficial for preventing or treating arthritis, cancer, cardiovascular disease, depression, developmental disabilities, diabetes, eye disease, hypertension, inflammatory disease, obesity and neurological disorders (Pan et al., 2012; Swanson et al., 2012). Usually, seed oils are the richest sources of α-linolenic acid, chia, perilla, flaxseed (linseed oil), rapeseed (canola) and soybeans a unique source such as thylakoid membranes in the leaves of *Pisum sativum* (pea leaves). The fact that micropropagation could enhance the alpha-linolenic acid contents in the shoots is certainly a significant breakthrough. Ertas et al. (2014) identified 10 fatty acids in field-grown plants of *Sedum sediforme* and the major fatty acids present in the whole plant extract were palmitic acid (28.8%), stearic acid (24.6%) and linolenic acid (12.9%). This variation may be owing to differences in the plant species and organ used for the extraction of FAMEs.

## Conclusions

Micropropagation of *S. dasyphyllum* has been demonstrated for the first time. Shoot multiplication was achieved best on MS medium incorporated with BA and NAA. The micropropagated shoots showed enhanced contents of all three bioactive compounds studied. HPLC-DAD and GC-MS analysis revealed the presence of four carotenoids, α-tocopherol and nine fatty acids in shoots. The high content of carotenoids, tocopherol, α-linolenic acid and linoleic acid in *S. dasyphyllum* encourage researchers to explore more this plant. Moreover, selective enrichment of the usually rare but popular fatty acid, alpha linolenic acid was observed in the micropropagated plants. Contents of lutein and α-tocopherol were higher in micropropagated shoots than the greenhouse-grown plants. Elicitation is reported to be used to increase the production and accumulation of secondary metabolites by *in vitro* production systems. Thus, appropriate elicitor treatments can possibly further enhance the *in vitro* production of bioactive compounds, more studies in this direction will lead to the ultimate exploitation of the rich reservoirs stored in this unexplored plant, *S. dasyphyllum*.

## Author contributions

HP, DK, and IS conceived, designed experiments, and writing the paper. RS and OL performed bioactive compounds analysis. JG and YK analyzed data. IS performed micropropagation research.

### Conflict of interest statement

The authors declare that the research was conducted in the absence of any commercial or financial relationships that could be construed as a potential conflict of interest.
